# Pulmonary aspiration as a biological process: inevitable and insufficiently explored

**DOI:** 10.1590/2317-1782/e20250097en

**Published:** 2025-11-17

**Authors:** Guilherme Maia Zica, Maria Inês Rebelo Gonçalves

**Affiliations:** 1 Universidade Federal de São Paulo – Unifesp - São Paulo (SP), Brasil.

Pulmonary aspiration is a biological process that occurs when solid or liquid particles enter the lower airways, that is, below the vocal folds. This mechanism can lead to outcomes ranging from no injury to death within a few minutes^(^1^)^. 

Reports of dysphagic patients have been published since the 1800s, first described by Helsham in *The Medical and Physical Journal* ^(^2^)^. Pulmonary aspiration began to be discussed in the scientific literature in 1937, invariably reported as an undesirable sequela of various comorbidities^(^3^)^. Evidently, the primary goal of any speech-language pathologist working with dysphagia is to minimize or prevent the occurrence of pulmonary aspiration^(^4^)^. Over the years, healthcare professionals, including speech-language pathologists, pulmonologists, and nurses, have sought to better understand its clinical repercussions and management^(^5^)^. 

In the literature, pulmonary aspiration is widely mentioned as a sequela or clinical finding secondary to comorbidities such as dysphagia^(^5,6^)^, gastrointestinal disorders^(^7^)^, tracheostomy^(^8,9^)^, and mechanical ventilation^(^10,11^)^. However, studies providing an in-depth description of pulmonary aspiration as a biological process, along with its clinical repercussions, findings, and parameters, are still lacking.

There are several questions regarding this mechanism that clinicians have yet to fully understand, such as: Is there a threshold considered “normal” for pulmonary aspiration? At what point does pulmonary aspiration become life-threatening? Is there any level of tolerance and/or protective mechanism in the body against pulmonary aspiration? Are some individuals more susceptible to complications? How can such a poorly understood—and often unavoidable—clinical finding be prevented or managed?

It is evident that there is still no definitive or unanimous answer to these questions in the scientific literature. Therefore, we aimed to organize, through specific categories, the findings reported in the literature in conjunction with our clinical and interprofessional experience to date, with the goal of questioning, reflecting, and stimulating interest, discussion, and research on pulmonary aspiration as a biological process.

## PREVALENCE

Understanding the prevalence of pulmonary aspiration is complex, as it is an underdiagnosed finding and difficult to measure. Studies describe it solely as a sequela of an underlying pathology, employing heterogeneous methodologies, and report conflicting results with inconclusive outcomes^(^3,4,6,8,9,12-14^)^. 

There are reports in the literature stating that clinical swallowing assessments may fail to detect aspiration in up to 50% of cases. Professional training and experience may influence this finding. Conversely, objective or instrumental examinations are described as more sensitive; however, they represent only a snapshot of function at a given moment and have inherent limitations^(^15-17^)^. 

The most consistent data regarding the prevalence of pulmonary aspiration pertain to patients following a stroke, with 2% to 25% (reaching up to 40% in some reports) of patients potentially experiencing aspiration^(^17,18^)^. The incidence of aspiration pneumonia in this population is high, ranging from 30% to 50% among dysphagic patients, but can be reduced to 13% in those with dysphagia who receive tube feeding^(^1^)^. 

Retrospective studies have shown that 20% to 30% of neurological and head-and-neck cancer patients with dysphagia may experience aspiration^(^16,17^)^. Individuals with non-progressive encephalopathy in childhood may present with severe aspiration throughout their lives^(^16^)^. 

Recent studies have reported a prevalence of up to 37% of pulmonary aspiration in patients undergoing extended partial laryngectomies during outpatient follow-up, even in the absence of clinical symptoms^(^4,19^)^. 

Understanding the prevalence of pulmonary aspiration can facilitate a more appropriate and careful approach in patients within at-risk contexts and groups. The definition and establishment of more structured parameters are essential for case management, particularly for less experienced professionals. The consolidation of risk and management guidelines for pulmonary aspiration is necessary, given that, in some cases, its occurrence is unavoidable.

## ETIOLOGY

To date, research has not provided clear evidence identifying which patients are at greatest risk of aspiration^(^15^)^. It is known that patients with swallowing disorders of various etiologies, upper and lower gastrointestinal disorders, tracheostomy, and mechanical ventilation are at increased risk of pulmonary aspiration^(^2,5,7,17^)^. 

We believe that, in the future, with advances in research and in technologies for the assessment and diagnosis of dysphagia, additional etiological factors for pulmonary aspiration will be identified. For example, a preliminary 2025 study reports the possibility of postoperative dysphagia following carotid endarterectomy in intensive care units—an aspect that previously would not have been considered among the risk criteria for swallowing disorders in the hospital setting^(^20^)^. 

Some authors report a higher risk of pulmonary aspiration in older adults over 80 years of age^(^6,19^)^. This finding suggests that certain elderly individuals may already present an increased risk even before a neurological or structural event occurs^(^6,21^)^. 

The mechanisms associated with pulmonary aspiration may include: reduced tongue pressure and strength^(^6^)^; central or localized weakness and incoordination of the pharyngeal musculature^(^5^)^; diminished pharyngeal and laryngeal sensitivity^(^9,11^)^; reduced laryngeal mobility^(^4,5,17^)^; deficits in swallowing sequencing^(^22^)^; delayed laryngeal closure^(^6^)^; reduced anterior movement of the hyoid bone^(^5,6^)^; ineffective reflexive coughing^(^10,23^)^; esophageal dysmotility^(^7^)^; and low levels of substance P or dopamine^(^5^)^. 

Drops in oxygen saturation are not always associated with aspiration. However, a low baseline oxygen saturation level (<94% SpO_2_) may be associated with an increased risk of aspiration^(^6^)^. 

Consolidated data regarding the different types of food and their association with aspiration risk are not yet available. It is known, however, that the greater the contamination rate and volume of aspirated residues, the higher the risk of pulmonary complications^(^5,6,16^)^. 

It is important to understand that swallowing is a complex neuromotor process with individual anatomical and physiological variations. Therefore, what may lead to aspiration is the presence—and, more importantly, the combination—of these factors within the individual and their context^(^21^)^. Isolated factors may not be predictive of aspiration.

By developing a more detailed and comprehensive understanding of the etiologies of aspiration, the interprofessional team can perform individualized, patient-centered mapping and prevention. This also enables the establishment of clearer criteria for determining the need for instrumental swallowing assessments and complementary evaluations.

## PROGNOSIS

The most common consequences of pulmonary aspiration include aspiration pneumonia, recurrent lower respiratory tract infections, and respiratory failure. Additionally, malnutrition and dehydration may serve as indicators. Patients may significantly reduce their intake of liquids and food, leading to weight loss^(^5,15-17^)^. 

Aspiration is not universally associated with increased morbidity and mortality in the literature^(^5^)^. The factors responsible for this finding are still not fully understood. Langmore^(^16^)^ suggests, regarding prognosis, that dysphagia and aspiration are risk conditions but are not sufficient on their own to cause pneumonia or functional decline^(^16^)^. Little is known about this assertion in the literature; however, it aligns with the practical experience of speech-language pathologists specializing in dysphagia.

The presence and severity of aspiration pneumonia may be related not only to the characteristics of the aspiration and the severity of dysphagia but also to numerous individual factors, such as the patient’s overall clinical condition, cognitive integrity, education, income/social class, mobility and independence in activities of daily living, pulmonary health, the volume and size of aspirated particles (macroaspiration and microaspiration), and the chemical composition and presence of infectious agents in the aspirated material^(^4,5,16,19,21,23^)^. 

In cases of emesis, it is widely accepted that gastric acid can cause chemical pneumonitis independently of bacterial infection, characterized by acute respiratory discomfort and hypoxia. Alternatively, in reflux, aspirated particles may mechanically accumulate and promote the growth of bacteria and fungi, leading to laryngeal irritation, acute or chronic respiratory discomfort, and an increased risk of secondary pneumonia^(^5^)^. 

None of the aspects mentioned above have well-defined and evidence-based standards in the literature. Much of the discussion on pulmonary aspiration is based on inferences from scientific studies and clinical observation. It is known that a minimally healthy lung is essential for adequately managing potential aspirations and maintaining bronchial hygiene. Patients with chronic obstructive pulmonary disease, asthma, or cystic fibrosis, for example, may be more susceptible to complications from aspiration^(^5,6,10^)^. 

It has been suggested that gravity may cause mucus, secretions, and aspirated material to accumulate inappropriately in the lungs when an individual remains lying down most of the time. Mobilization becomes necessary to promote bronchial hygiene. Pulmonary physiotherapy exercises and devices such as the Shaker also appear to assist in this process^(^10^)^. 

By carefully selecting the parameters that may influence the prognosis of patients with pulmonary aspiration, it may be possible to implement preventive interventions and adopt a more targeted management approach, aiming to minimize its impact on the patient’s health and quality of life, as well as that of their family.

For example, when necessary, clearing maneuvers can be recommended during and after feeding, as well as bronchial hygiene exercises, such as using the Shaker device after meals. It is possible to develop a therapeutic plan in collaboration with the respiratory physiotherapy team and the gastroenterologist, incorporating behavioral strategies—such as caregiver guidance—and management of the environment in which the patient eats.

## TREATMENT

In clinical practice, it is common to observe patients with dysphagia and signs of pulmonary aspiration who continue to receive oral feeding, either exclusively or in combination with other methods. Pulmonary aspiration is sometimes an unavoidable finding, in which case the speech-language pathologist, with the support of the interdisciplinary team, works to ensure that feeding is as safe, efficient, and comfortable as possible.

As is well known, speech-language pathology offers numerous strategies for managing swallowing, including orofacial myofunctional exercises, vocal exercises, facilitating maneuvers, and adjustments to food consistency, as well as the volume and speed of food and liquid administration. Additionally, there are tools for managing saliva, secretions, and gastrointestinal disorders. As noted, the presence of dysphagia and aspiration is not predictive of clinical or pulmonary complications, and their management may not necessarily prevent comorbidities. It is the responsibility of the care team to determine an individualized management plan.

The literature describes the potential identification of a breathy vocal quality as an indicator of pulmonary aspiration risk, as well as impairments in laryngeal sensitivity^(^24^)^. 

Studies indicate that some simple measures can be implemented in at-risk groups for aspiration, such as frail and institutionalized elderly patients. Oral cavity hygiene and maintaining an upright posture after meals are associated with reduced rates of respiratory infections^(^25^)^. 

Interprofessional collaboration enables the implementation of practices that prioritize quality of life, balancing the wishes of the patient and family with therapeutic limitations and possibilities. Continuous dialogue is essential regarding the benefits and risks of maintaining oral feeding in cases where aspiration is presumed or observed.

According to the data presented in this article, addressing dysphagia in isolation may not be effective in preventing aspiration pneumonia. It is understood that, in many cases, pulmonary aspiration is not resolved but rather managed.

There is still limited in-depth knowledge in the literature regarding pulmonary aspiration as a biological process. Interdisciplinary work is essential: nutrition aims to ensure adequate nutrient intake and hydration; physiotherapy supports overall mobility, metabolic reserve, and pulmonary rehabilitation; the physician focuses on maintaining clinical and pulmonary stability, potentially implementing prophylactic pharmacological measures to manage pulmonary aspiration; and the speech-language pathologist manages swallowing function.

Biological, prospective, and interdisciplinary studies are needed to understand the mechanisms of pulmonary aspiration and their impacts on the body and patient health, thereby enabling the development of clearer guidelines for determining appropriate, targeted, and individualized management strategies.
