# Supplementary material for: Pulmonary aspiration as a biological process: inevitable and insufficiently explored
Source: Codas. 2025 Nov 17;37(6):e20250097. doi: 10.1590/2317-1782/e20250097en (PMC12642775; doi:10.1590/2317-1782/e20250097en)
Supplement: Translation

# Translation: Aspiração pulmonar como processo biológico: por vezes inevitável e pouco compreendida

**DOI:** 10.1590/2317-1782/e20250097pt

**Authors:** Guilherme Maia Zica, Maria Inês Rebelo Gonçalves

**Affiliations:** 1 Universidade Federal de São Paulo – Unifesp - São Paulo (SP), Brasil.

A aspiração pulmonar é um processo biológico que ocorre quando partículas sólidas ou líquidas entram para o interior das vias aéreas inferiores, ou seja, abaixo das pregas vocais. Esse mecanismo pode ter consequências que variam de nenhuma lesão até a morte em poucos minutos^(1)^. 

Há relatos de pacientes disfágicos desde 1800, publicados e descritos por Helsham no *The Medical and Physical Journal* ^(2)^ *.* A aspiração pulmonar começou a ser discutida na literatura em 1937, sempre como sequela indesejada de diferentes comorbidades^(3)^. Evidentemente, o objetivo fundamental de qualquer fonoaudiólogo que atue em disfagia é minimizar ou evitar a ocorrência da aspiração pulmonar^(4)^. Ao longo dos anos, profissionais da área da saúde como fonoaudiólogos, pneumologistas e enfermeiros tentaram compreender melhor suas repercussões clínicas e seu manejo^(5)^. 

Na literatura, a aspiração pulmonar é amplamente mencionada como sequela ou achado clínico secundário a uma comorbidade como disfagia^(5,6)^, distúrbios gastrointestinais^(7)^, traqueostomia^(8,9)^ e ventilação mecânica^(10,11)^. Porém, ainda não encontramos estudos que a descreva com profundidade como um processo biológico e suas repercussões, achados e parâmetros clínicos.

Existem diversos questionamentos sobre esse mecanismo que os clínicos ainda não conseguem compreender, como: existe um limite considerado “normal” para ocorrência de aspiração pulmonar? Em que ponto seria considerada um risco para vida? Existe algum nível de tolerância e/ou mecanismo de defesa do organismo para esse distúrbio? Algumas pessoas são mais suscetíveis a complicações? Como prevenir e/ou controlar um achado clínico tão pouco compreendido e, por muitas vezes, inevitável?

É evidente que ainda não existe uma resposta correta ou unânime para todos esses questionamentos na literatura científica. Assim, buscamos organizar por meio de categorias o que encontramos na literatura em associação com nossa experiência clínica e interprofissional até o momento, visando questionar, refletir, incentivar e direcionar o interesse, discussões e pesquisas sobre a aspiração pulmonar como um processo biológico.

## PREVALÊNCIA

Compreender a prevalência da aspiração pulmonar é algo complexo, por vezes um achado subdiagnosticado e de difícil mensuração. Os estudos a descrevem somente como sequela de uma patologia de base por meio de metodologias heterogêneas e apresentam resultados conflitantes e desfechos pouco conclusivos^(3,4,6,8,9,12-14)^.

Existem relatos na literatura que afirmam que a avaliação clínica da deglutição pode não detectar a aspiração em até 50% dos casos. Podemos presumir que a formação e a experiência profissional podem influenciar neste achado. Em contrapartida, descrevem que os exames objetivos / instrumentais são mais sensíveis, porém, representam um recorte temporal da função naquele momento e apresentam limitações^(15-17)^.

Os dados mais consistentes em relação à prevalência da aspiração pulmonar são para pacientes após acidente vascular cerebral, no qual de 2% a 25% (chegando até a 40% em alguns relatos) dos pacientes podem apresentar aspiração^(17,18)^. A frequência de pneumonia aspirativa neste grupo é alta, variando de 30% a 50% em pacientes disfágicos, podendo ser reduzida para 13% em pacientes com disfagia que utilizam sonda para alimentação^(1)^. 

Estudos retrospectivos mostraram que de 20% a 30% dos pacientes disfágicos neurológicos e com câncer de cabeça e pescoço podem aspirar^(16,17)^. Indivíduos com encefalopatia não progressiva da infância podem apresentar aspirações graves ao longo de toda a vida^(16)^. 

Pesquisas recentes relataram prevalência de até 37% de aspiração pulmonar em pacientes submetidos a laringectomias parciais alargadas em controle ambulatorial e sem sintomas clínicos^(4,19)^. 

Compreender a prevalência desse distúrbio pode favorecer uma abordagem mais adequada e cuidadosa em pacientes em contextos e grupos de risco. A definição e construção de parâmetros mais estruturados é fundamental na condução dos casos, especialmente para profissionais menos experientes. É necessária a consolidação de norteadores de risco e manejo da aspiração pulmonar, uma vez que sabemos que, em alguns casos, sua ocorrência é inevitável.

## ETIOLOGIA

Pesquisas até o momento não demonstraram evidências claras para indicar quais pacientes apresentam maior risco de aspiração^(15)^. Sabe-se que pacientes com transtornos de deglutição (de diferentes etiologias), distúrbios gastrointestinais (altos e baixos), traqueostomia e ventilação mecânica apresentam maior risco para ocorrência de aspiração pulmonar^(2,5,7,17)^. 

Acreditamos que a tendência no futuro, com o avanço das pesquisas e tecnologias de avaliação e diagnóstico da disfagia, é descobrir ainda novos fatores etiológicos da aspiração pulmonar. Por exemplo, um estudo preliminar de 2025 relata a possibilidade de disfagia no pós operatório de endarterectomia de carótida em unidades de terapia intensiva. Informação essa que, anteriormente, não seria levada em consideração nos critérios de risco para transtornos de deglutição no ambiente hospitalar^(20)^. 

Alguns autores referem maior risco de aspiração pulmonar em idosos acima de 80 anos^(6,19)^. Esse achado sugere que alguns idosos podem já apresentar risco aumentado antes mesmo de um evento neurológico ou estrutural^(6,21)^. 

Os mecanismos associados à aspiração pulmonar podem incluir: pressão e força de língua reduzidas^(6)^, fraqueza e incoordenação central ou local da musculatura faríngea^(5)^, sensibilidade faríngea e laríngea reduzidas^(9,11)^, mobilidade laríngea reduzida^(4,5,17)^, déficits no sequenciamento da deglutição^(22)^, atraso do fechamento laríngeo^(6)^, movimento anterior do osso hióide reduzido^(5,6)^, tosse reflexa ineficaz^(10,23)^, dismotilidade do esôfago^(7)^ e baixos níveis de substância P ou dopamina^(5)^. 

Quedas na saturação de oxigênio nem sempre estão associadas à aspiração. No entanto, um baixo nível basal de saturação de oxigênio (<94% SpO2) pode estar associado ao aumento do risco de aspiração^(6)^. 

Ainda não existem dados consolidados em relação aos diferentes tipos de alimento e ao risco de aspiração. Sabe-se que, quanto maior a taxa de contaminação dos resíduos aspirados e seu volume, maior o risco de complicações pulmonares^(5,6,16)^. 

É preciso compreender que a deglutição é um processo neuromotor complexo com variações anatômicas e fisiológicas individuais e, portanto, o que pode levar a aspiração é a presença e, principalmente, a associação destes fatores no indivíduo e seu contexto. ^(21)^ Fatores isolados podem não ser preditivos para aspiração.

Ao compreender com maior detalhe e complexidade as etiologias da aspiração, a equipe interprofissional pode realizar um trabalho de mapeamento e prevenção individualizado focado nos pacientes. Além disso, torna-se possível estabelecer critérios mais claros para a deliberação de exames instrumentais da deglutição e avaliações complementares.

## PROGNÓSTICO

As consequências mais comuns da aspiração pulmonar incluem pneumonia por aspiração, infecções por repetição do trato respiratório inferior e insuficiência respiratória. Além disso, desnutrição e desidratação podem ser indicadores. Os pacientes podem reduzir consideravelmente sua ingestão de líquidos e alimentos e perder peso^(5,15-17)^.

A aspiração não é associada ao aumento da morbidade e mortalidade de forma unânime na literatura^(5)^. A compreensão dos fatores responsáveis por esse achado ainda não é clara. Langmore^(16)^ em 1998 sugere, em relação ao prognóstico, que a disfagia e aspiração são condições de risco mas não suficientes para o desenvolvimento de pneumonia e declínio funcional^(16)^. Pouco se sabe sobre essa afirmação, porém, é um relato verdadeiro na prática do fonoaudiólogo especialista em disfagia.

A presença e a gravidade da pneumonia broncoaspirativa pode estar relacionada não somente à característica da aspiração/ disfagia mas sim a inúmeras caraterísticas individuais como: o estado clínico geral do paciente, integridade cognitiva, escolaridade e renda, mobilidade e autonomia, saúde e integridade pulmonar, volume e tamanho das partículas aspiradas (macroaspiração e microaspiração) e composição química e presença de agentes infecciosos do material aspirado^(4,5,16,19,21,23)^. 

Em casos de êmese, já é consenso que o ácido gástrico pode causar uma pneumonite química independente de infecção bacteriana, caracterizada por desconforto respiratório agudo e hipóxia. Alternativamente, no refluxo, partículas aspiradas podem se acumular mecanicamente e favorecer o desenvolvimento de bactérias e fungos, causando desconforto laríngeo, respiratório agudo ou crônico e aumentando o risco de pneumonia secundária^(5)^. 

Nenhum destes aspectos citados possui padrões descritos com detalhes e comprovados na literatura. Muito do que se discute em aspiração pulmonar é pautado em inferências de estudos científicos e observação clínica. Sabe-se que é imprescindível um pulmão minimamente saudável para gerenciar adequadamente as possíveis aspirações e realizar sua higiene brônquica. Pacientes com doença pulmonar obstrutiva crônica, asma e fibrose cística, por exemplo, podem ser mais suscetíveis a complicações da aspiração^(5,6,10)^. 

Sugere-se que a influência da gravidade pode fazer com que o muco, a secreção e o material aspirado se acumule de forma inadequada nos pulmões quando o indivíduo apresenta-se na maior parte do tempo deitado. A mobilização torna-se necessária com o objetivo de favorecer a higiene brônquica. Exercícios de fisioterapia pulmonar e aparelhos como o Shaker também parecem auxiliar nesse processo^(10)^. 

Elegendo de forma criteriosa os parâmetros que podem influenciar no prognóstico dos pacientes que apresentam aspiração pulmonar, talvez seja possível realizar uma intervenção preventiva e uma condução mais assertiva, a fim de minimizar suas consequências na saúde e qualidade de vida do paciente e sua família.

Por exemplo, quando necessário, podemos sugerir manobras de limpeza durante e após as refeições e realização de exercícios de higiene brônquica como o aparelho Shaker após a alimentação. É possível desenvolver um planejamento terapêutico em conjunto com equipe de fisioterapia respiratória e médico gastroenterologista, associando a modulação de aspectos comportamentais como orientações a cuidadores e questões subjetivas como o contexto alimentar.

## TRATAMENTO

Durante a prática clínica é comum observar pacientes com quadros de disfagia e sinais de aspiração pulmonar que permanecem se alimentando por via oral de forma exclusiva ou não. A aspiração pulmonar, por vezes, é um achado inevitável, no qual o fonoaudiólogo, com auxílio da equipe interdisciplinar, trabalha na tentativa de manter a alimentação da forma mais segura, eficiente e confortável possível.

Como sabemos, a fonoaudiologia possui muitas estratégias para o gerenciamento da deglutição, como exercícios oromiofuncionais,exercícios vocais, manobras facilitadoras e ajustes de consistência, volume e velocidade de oferta dos alimentos e líquidos. Além disso, existem ferramentas para controle de saliva, secreções e transtornos gastrointestinais. Como descrito, a presença de disfagia e aspiração não é preditivo para complicações clínicas e pulmonares, assim como, seu manejo pode necessariamente não impedir comorbidades. Cabe à equipe de cuidado a deliberação de uma condução individualizada.

A literatura descreve a possibilidade da identificação de uma qualidade vocal soprosa para risco de aspiração pulmonar, assim como, comprometimentos de sensibilidade laríngea^(24)^. 

Estudos afirmam que algumas medidas simples podem ser realizadas em grupos de risco para aspiração, como em pacientes idosos frágeis e institucionalizados. A limpeza da cavidade oral e manutenção de uma postura ereta após as refeições apresenta correlação com a redução das taxas de infecções respiratórias^(25)^. 

O trabalho interprofissional viabiliza a deliberação de condutas pautadas sempre na manutenção da qualidade de vida, ponderando o desejo do paciente, da família e os limites e possibilidades terapêuticas. É preciso diálogo constante sobre os benefícios e malefícios da manutenção da dieta por via oral em alguns casos com presença de aspiração presumida ou observada.

Conforme os dados descritos, cuidar somente da disfagia pode não ser eficaz na prevenção de pneumonia broncoaspirativa. Entende-se que, em muitos casos, a aspiração pulmonar não é solucionada, mas sim, gerenciada.

Ainda não existe um conhecimento profundo na literatura acerca da aspiração pulmonar como processo biológico. Um trabalho interdisciplinar é essencial: a nutrição tenta garantir nutrientes e hidratação; a fisioterapia auxilia na mobilidade global, reserva metabólica e reabilitação pulmonar; o médico busca controlar a saúde/estabilidade clínica e pulmonar, sendo possível utilizar algumas medidas profiláticas; e o fonoaudiólogo realiza o gerenciamento da deglutição.

São necessários estudos biológicos, prospectivos e interdisciplinares para compreender os mecanismos da aspiração pulmonar e seus impactos no corpo e na saúde dos pacientes e, assim, possibilitar a formulação de norteadores com maior clareza para deliberação de condutas e estratégias assertivas e individualizadas.
